# Association of *EPHA2* polymorphisms and age-related cortical cataract in a Han Chinese population

**Published:** 2011-06-09

**Authors:** Wei Tan, Shengping Hou, Zhengxuan Jiang, Zheng Hu, Peizeng Yang, Jian Ye

**Affiliations:** 1 Department of Ophthalmology, Research Institute of Field Surgery, Da Ping Hospital, Third Military Medical University, Chongqing, P.R. China; 2 The First Affiliated Hospital of Chongqing Medical University, Chongqing, P.R. China; 3 Chongqing Key Laboratory of Ophthalmology, Chongqing, P.R. China

## Abstract

**Purpose:**

The gene for Eph-receptor tyrosinekinase-type A2 (*EPHA2*) has been shown to be involved in the pathogenesis of age-related cataract (ARC). The aim of this study was to examine whether *EPHA2* polymorphisms were associated with the susceptibility to age-related cortical cataract in a Han Chinese population.

**Methods:**

Five single-nucleotide polymorphisms (SNPs)—rs3768293, rs3754334, rs7548209, rs707455, and rs477558—in the *EPHA2* gene were genotyped in 422 Han Chinese patients with age-related cortical cataract and 317 age-, sex-, and ethnically matched healthy controls using a PCR restriction fragment length polymorphism (PCR-RFLP) assay. Data were analyzed by χ^2^ analysis.

**Results:**

The results showed that the five analyzed polymorphisms in *EPHA2* were in Hardy–Weinberg equilibrium both in the patients and in the controls. The frequency of the rs477558 AA genotype was signiﬁcantly increased in ARC patients compared with controls (χ^2^=8.649, pc=0.045, odds ratio [OR] 1.555, 95% CI 1.158 to 2.089). The frequency of the rs477558 AG genotype was signiﬁcantly decreased in ARC patients compared with controls (χ^2^=9.281, pc=0.030, OR 0.626, 95% CI 0.463 to 0.847). Signiﬁcantly higher frequencies of the GG genotype and the G allele of rs7548209 were observed in ARC patients compared with controls (χ^2^=10.430, pc=0.015, OR 1.660, 95% CI 1.219 to 2.261 and χ^2^=8.537, pc=0.015, OR 1.486, 95% CI 1.138 to 1.940, respectively). On the other hand, signiﬁcantly decreased frequencies of the rs7548209 CG genotype and the C allele were observed in ARC patients compared with controls (χ^2^=9.999, pc=0.030, OR 0.603, 95% CI 0.440 to 0.826 and χ^2^=8.537, pc=0.015, OR 0.673, 95% CI 0.515 to 0.879, respectively). There was no difference in the frequencies of the genotype and allele of the rs3768293, rs3754334, and rs707455 SNPs between the patients with ARC and the controls.

**Conclusions:**

Our study suggests that both SNP rs477558 and SNP rs7548209 of *EPHA2* are associated with age-related cortical cataract in a Han Chinese population.

## Introduction

Cataract is the leading cause of visual impairment worldwide, with approximately 37 million people affected, accounting for 48% of global blindness and with approximately half of all cases originating in Africa and Asia [1,2]. Approximately 80% of all cataracts are age-related and idiopathic [3]. Age-related cataract (ARC) has been deﬁned as the appearance of the clinical sign of cataracts in one or both eyes in a person older than 50 years [4]. ARC accounts for 18 million cases of blindness and 59 million cases of reduced vision worldwide [5]. Depending on the location of the opacity within the lens, age-related cataracts can be divided into four categories: nuclear, cortical, posterior subcapsular, and mixed type [6]. The development of ARC is complex and multi-factorial, where genetic predisposition and environmental elements both contribute to the pathological condition. Family epidemiological investigations have showed that hereditary factors play an important role in the occurrence of ARC [7-11]. Moreover, it is known that cortical cataract is highly heritable among four types of age-related cataract [10,11]. Over the past several years, to have better comprehension of the molecular processes that are associated with cataract development, attempts have been made to identify the genes and characterize the proteins they encode [3,12-18]. However, the exact genetic mechanism and pathogenesis of age-related cataract remain unclear.

Eph-receptor tyrosinekinase-type A2 (*EPHA2*) encodes a 976 amino acid, type-1 transmembrane protein with extracellular NH_2_-terminal and cytoplasmic COOH-terminal halves [19] and is a member of the largest sub-family of receptor tyrosine kinases. The human *EPHA2* gene is located on chromosome 1p36, where linkage with autosomal dominant and autosomal recessive cataracts has been reported [20-24]. Recently, several *EPHA2* variants were found to be associated with age-related cortical cataracts in different worldwide Caucasian populations [5,13,25]. Furthermore, accumulated evidences have shown that genetic heterogeneity existed in different ethnic cohorts. Therefore, we examined whether *EPHA2* polymorphisms were associated with the susceptibility to age-related cataract in a Han Chinese population. In this study we found that single-nucleotide polymorphisms (SNPs) rs477558 and rs7548209 in *EPHA2* were associated with age-related cortical cataract in a Han Chinese population.

## Methods

### Subjects

The study adhered to the tenets of the Declaration of Helsinki. All participants signed the respective informed consent forms. The research was approved by the Ethics Committee of Research Institute of Field Surgery, Da Ping Hospital, Third Military Medical University, Chongqing, P.R. China. The subjects were 422 sporadic Han Chinese patients with age-related cortical cataracts and 317 normal controls. All subjects were unrelated Chinese individuals recruited from the Research Institute of Field Surgery, Da Ping Hospital, Third Military Medical University, Chongqing, P.R. China, from March 2010 to March 2011. Patients with secondary cataracts caused by persistent intraocular inflammation, trauma, uveitis, high myopia, glaucoma, or degenerated ocular diseases were excluded from this study. Information on hypertension, diabetes mellitus, prolonged corticosteroid administration, and other known causes were not available. Controls were individuals who visited the same hospital for a routine ophthalmic examination and were age-, sex-, and ethnically matched with the ARC patients. The control group included unrelated healthy individuals without history of cataract, hypertension, diabetes, tumor, or other ocular diseases. Baseline characteristics of study participants are shown in Table 1. All ARC patients and control subjects underwent a full ophthalmic examination, including visual acuity, lens examination in transient and side illumination with a slit lamp biomicroscope after mydriasis, and ophthalmoscopic examination. The degrees of cataract in all patient eyes were CII or CIII according to the Lens Opacities Classification System, version II (LOCS II) [26]. The degrees of nuclear hardness in all eyes were equal to or more than grade III according to the Emery and Little nuclear hardness classification. All the patients then received phacoemulsification and intraocular lens implantation surgery.

**Table 1:** Baseline characteristics of study participants.

** **	** **	**Sex**		**Age (years)**	
**Group**	**n**	**Male n (%)**	**Female n (%)**	**Mean±SD**	**Range**
ARC patients	422	183 (43.36)	239 (56.64)	63.58±6.76	50–89
Controls	317	149 (47.00)	168 (53.00)	61.92±6.03	50–83

ARC, age-related cortical cataract

### Blood samples and DNA extraction

Venous blood samples were obtained from the ARC patients and controls in EDTA Vacutainers (BD, Franklin Lakes, NJ). Genomic DNA was extracted from peripheral venous blood using a QIAamp DNA Blood Mini Kit (Qiagen, Hilden, Germany) according to the manufacturer’s instructions. DNA samples were collected in 1.5 ml Eppendorf tubes and stored at −20 °C until used. DNA concentrations were measured by NanoDrop 2000 Spectrophotometer (NanoDrop Technologies, Wilmington, DE).

### SNP selection and genotyping

The SNPs were selected from earlier studies on the association of *EPHA2* polymorphisms with age-related cataract in other ethnic groups [5,13]. We therefore selected rs3768293, rs3754334, rs477558, rs707455, and rs7548209 as candidate SNPs. Ampliﬁcation of the target DNA in *EPHA2* was analyzed by polymerase chain reaction (PCR) using primers presented in Table 2. Each PCR reaction was performed in a 10 μl reaction mixture containing 5 μl Premix Taq (Ex Taq Version; TaKaRa Biotechnology Co. Ltd., Dalian, China), 20 pmol primers, and 0.2 μg of genomic DNA for ampliﬁcation of the DNA. The conditions were as follows: initial denaturation at 95 °C for 5 min followed by 38 cycles of denaturation at 94 °C for 30 s, annealing at different temperatures (54 °C for rs3768293 and rs3754334, 56 °C for rs477558, and 60 °C for rs707455 and rs7548209) for 30 s, extension at 72 °C for 30 s, and a ﬁnal extension at 72 °C for 5 min. These SNPs were genotyped by PCR-restriction fragment length polymorphism analysis. PCR products of rs3768293, rs3754334, rs477558, rs707455, and rs7548209 polymorphisms were respectively digested with 4 U of Tru1I (MBI Fermentas, Burlington, ON, Canada), TasI (MBI Fermentas), AluI (Promega, Madison, WI), HpyF10VI (Promega), and Hsp92II(Promega) restriction enzymes (Table 2) in a 10 μl reaction volume overnight. Digestion products were visualized on a 4.0% agarose gel and stained with GoldView (SBS Genetech, Beijing, China). Direct sequencing was performed by Invitrogen Biotechnology Company (Shanghai, China) using the randomly selected subjects (20% of all samples) to validate the method used in this study.

**Table 2:** Primers and restriction enzymes used for RFLP analysis of the *EPHA2* gene.

**rs number**	**Primers**	**Restriction enzyme**
rs3768293	5′-CAGCTTTCTGGAGTCTCAGTTTTATT-3′	Tru1I
	5′-TCCCTACGTTATATGAGTACCC-3′	
rs3754334	5′-CCCGGCCACCAGAAGCGCAT-3′	TasI
	3′-GACCTCGGGGTAGCCGGTTCTT-5′	
rs477558	5′-ATAAAGATGTGGCTAGAGGT-3′	AluI
	3′-TGCGTAAGTCGTTTCCACCT-5′	
rs707455	5′-CTGATGTAGATGGGGTGCTG-3′	HpyF10VI
	3′-TTTTACCTACACTCTCCGAC-5′	
rs7548209	5′-GGAGAATGGTTTTTAGGAAGCAACAT-3′	Hsp92II
	3′-TTATTTCAAAGGGGAGGACGGT-5′	

### Statistical analysis

Hardy–Weinberg equilibrium was tested using the χ^2^ test. The number of genotypes and alleles were obtained by direct counting. Allele and genotype frequencies were compared between ARC patients and controls by the χ^2^ test using SPSS (version 17.0, SPSS, Inc., Chicago, IL). The p values were corrected (pc) with the Bonferroni correction by multiple comparisons with the numbers of analyses performed. Statistical significance was reached if pc<0.05.

## Results

The results showed that the five analyzed *EPHA2* genetic variants were in Hardy–Weinberg equilibrium in both the ARC patients and the controls. No difference was found in the distribution of the age and gender ratio between ARC patients and controls. The distributions of genotypic frequencies and allelic frequencies of the five tested *EPHA2* polymorphisms are shown in Table 3.

**Table 3:** Frequencies of genotypes and alleles of *EPHA2* polymorphisms in ARC patients and controls.

**SNP**	**Genotype allele**	**ARC (%) (n=422)**	**Controls (%) (n=317)**	**χ^2^**	**p value**	**pc value**	**OR (95% CI)**
rs3768293	AA	314 (74.41)	210(66.25)	5.845	0.016	NS	1.481 (1.076~2.039)
	AC	96 (22.75)	99 (31.23)	6.704	0.010	NS	0.648 (0.467~0.901)
	CC	12 (2.84)	8 (2.52)	0.070	0.791	NS	1.130 (0.457~2.799)
	A	724 (85.78)	519 (81.86)	4.162	0.041	NS	1.337 (1.011~1.768)
	C	120 (14.22)	115 (18.14)	4.162	0.041	NS	0.748 (0.566~0.989)
rs3754334	CC	301 (71.33)	222 (70.03)	0.147	0.702	NS	1.065 (0.773~1.466)
	CT	111 (26.30)	84 (26.50)	0.004	0.953	NS	0.990 (0.711~1.378)
	TT	10 (2.37)	11 (3.47)	0.794	0.373	NS	0.675 (0.283~1.610)
	C	713 (84.48)	528 (83.28)	0.386	0.534	NS	1.093 (0.826~1.445)
	T	131 (15.52)	106 (16.72)	0.386	0.534	NS	0.915 (0.692~1.211)
rs477558	AA	260 (61.61)	161 (50.79)	8.649	0.003	0.045	1.555 (1.158~2.089)
	AG	135 (31.99)	136 (42.90)	9.281	0.002	0.030	0.626 (0.463~0.847)
	GG	27 (6.40)	20 (6.31)	0.002	0.961	NS	1.015 (0.558~1.845)
	A	655 (77.61)	458 (72.24)	5.607	0.018	NS	1.332 (1.050~1.689)
	G	189 (22.39)	176 (27.76)	5.607	0.018	NS	0.751 (0.592~0.952)
rs707455	TT	218 (51.66)	183 (57.73)	2.687	0.101	NS	0.782 (0.583~1.049)
	CT	178 (42.18)	122 (38.48)	1.024	0.311	NS	1.166 (0.866~1.570)
	CC	26 (6.16)	12 (3.79)	2.094	0.148	NS	1.669 (0.829~3.361)
	T	614 (72.75)	488 (76.97)	3.404	0.065	NS	0.799 (0.629~1.014)
	C	230 (27.25)	146 (23.03)	3.404	0.065	NS	1.252 (0.986~1.590)
rs7548209	GG	302 (71.56)	191 (60.25)	10.430	0.001	0.015	1.660 (1.219~2.261)
	CG	110 (26.07)	117 (36.91)	9.999	0.002	0.030	0.603 (0.440~0.826)
	CC	10 (2.37)	9 (2.84)	0.159	0.690	NS	0.831 (0.333~2.069)
	G	714 (84.60)	499 (78.71)	8.537	0.003	0.015	1.486 (1.138~1.940)
	C	130 (15.40)	135 (21.29)	8.537	0.003	0.015	0.673 (0.515~0.879)

OR, odds ratio; pc, Bonferroni corrected p value; NS, not significant.

The results showed that there were signiﬁcant differences between the ARC patients and the controls in the genotype or allele frequencies of rs477558 and rs7548209. The frequency of the AA genotype of rs477558 was signiﬁcantly increased in ARC patients compared with controls (χ^2^=8.649, pc=0.045, odds ratio [OR] 1.555, 95% CI 1.158 to 2.089). The frequency of the rs477558 AG genotype was signiﬁcantly decreased in ARC patients compared with controls (χ^2^=9.281, pc=0.030, OR 0.626, 95% CI 0.463 to 0.847). Signiﬁcantly higher frequencies of the GG genotype and the G allele of rs7548209 were observed in ARC patients compared with controls (χ^2^=10.430, pc=0.015, OR 1.660, 95% CI 1.219 to 2.261 and χ^2^=8.537, pc=0.015, OR 1.486, 95% CI 1.138 to 1.940, respectively). On the other hand, signiﬁcantly decreased frequencies of the rs7548209 CG genotype and the C allele were observed in ARC patients compared with controls (χ^2^=9.999, pc=0.030, OR 0.603, 95% CI 0.440 to 0.826 and χ^2^=8.537, pc=0.015, OR 0.673, 95% CI 0.515 to 0.879, respectively).

Increased frequencies of the AA genotype and the A allele of rs3768293 were also observed in patients with ARC compared with normal controls (χ^2^=5.845, p=0.016, OR1.481, 95%CI 1.076 to 2.039 and χ^2^=4.162, p=0.041, OR1.337, 95% CI 1.011 to 1.768, respectively). However, the difference was lost when the Bonferroni correction was performed (pc=0.240 and pc=0.205, respectively), and there was no difference in the frequencies of the rs3754334 and rs707455 SNPs between the ARC patients and the controls.

Haplotype analysis was performed by SHEsis platform [27], and no significant association was found between haplotype and ARC.

## Discussion

In this study we focused on the polymorphisms of *EPHA2*, which have been shown to affect its expression level and the clinical presentation of ARC [5,13], and we performed a case-control association study for these polymorphisms with ARC in a Han Chinese population. The results showed that SNP rs477558 and SNP rs7548209 were associated with the susceptibility to age-related cortical cataract.

EPHA2 belongs to the largest sub-family of receptor tyrosine kinases, and the Eph receptors and their membrane-anchored ephrin ligands form cell-contact-dependent bidirectional signaling pathways affecting diverse cellular processes including: actin cytoskeleton, cell-substrate adhesion, cell shape and cell movement, cell proliferation, survival, and differentiation [28-31]. Previous studies have shown that the EPH/ephrin system is critical for vision processes in the midbrain [32] and for neural development [33], and it was recently reported that ephrin-A5, a ligand of EPHA2, acts as a regulator for EPHA2, and loss of ephrin-A5 function can lead to cataract by disrupting lens fiber cell-packing in mice [34]. In 2009 [5],  it was demonstrated that cytoplasmic trapping was likely to interrupt both signaling and structural functions of EPHA2/ephrinA system in lens fiber cells. Furthermore, cataractogenesis in *EphA2* knockout mice implied that *EphA2* was essential for maintaining lens clarity [5]. In addition, genetic variants of the *EPHA2* gene were identified to confer risks for age-related cortical cataracts in a Caucasian population [5]. The association of *EPHA2* with age-related cataracts in Caucasians prompted us to investigate its association with ARC in the Han Chinese population.

Our results showed that the rs7548209 SNP was positively associated with ARC in the Han Chinese population. This result is consistent with earlier findings of ARC studies in three worldwide Caucasian populations [5]. We also detected an association between the rs477558 SNP and ARC, a result which is in agreement with earlier findings reported in an ARC study of a Northern Italian population [13]. Meta-analysis showed a strong association of rs3768293 and rs3754334 in the *EPHA2* gene with ARC in three worldwide Caucasian populations [5]. In addition, rs707455 has been reported to be associated with ARC in the Northern Italian population [13]. Our study failed to find an association of the aforementioned three SNPs with ARC. On the other hand, two SNPs in *EPHA2*, rs6603867 and rs6678616, have been investigated for their significant association with ARC in the different Caucasian groups [5]. As rs6678616 is not polymorphic and the minor allele frequency of rs6603867 is low (5%) in the Han Chinese population according to the International HapMap data, they were ﬁnally excluded in this study. Taken together, these results suggest that SNP-based allele frequencies and thereby association with a disease phenotype often vary among different ethnic groups.

As association studies may be influenced by many factors, the following measures were used to validate the results. First, only patients with primary cortical cataracts who underwent lens extraction were included in the present study. If there was any doubt, patients were excluded from the study. Then, unrelated healthy individuals were selected from the same geographical regions as the ARC patients. The patients and controls were age-, sex-, and ethnically matched. Finally, 20% of the samples were randomly chosen and analyzed by direct sequencing in an attempt to validate the PCR-RFLP data.

As with other candidate gene studies, there are several limitations in our study. First, as the power to detect disease susceptibility genes is influenced by the number of patient samples, the size of the patient sample group in our study seemed to be relatively small, and the patients were recruited only from the Han Chinese population. The results observed in this study need to be confirmed using larger sample sizes and other ethnic populations. Second, the biologic functions of rs477558 and rs7548209, two associated SNPs demonstrated in our study, need to be further investigated. Third, age-related cataract is termed as nuclear, cortical, posterior subcapsular, or mixed cataract by the zone of opacification. In this study, we included only cortical cataract cases. The association of SNPs in *EPHA2* might account for all types of ARC, so our study also needs to detect the association of *EPHA2* with the other three types.

In conclusion, our study identiﬁed the association of rs477558 and rs7548209 in *EPHA2* with age-related cortical cataract in a Han Chinese population. A large number of well matched samples, multiple ethnic groups, and further functional studies will contribute to the understanding of genetic predisposition to risk of ARC, identiﬁcation of disease-causing variants, and knowledge of the underlying mechanisms by which disease-causing variants affect disease susceptibility. To our knowledge, the present study is the first report of a Han Chinese population on the association of *EPHA2* with age-related cortical cataract.
